# A Chromosome 7 Pericentric Inversion Defined at Single-Nucleotide Resolution Using Diagnostic Whole Genome Sequencing in a Patient with Hand-Foot-Genital Syndrome

**DOI:** 10.1371/journal.pone.0157075

**Published:** 2016-06-07

**Authors:** Christopher M. Watson, Laura A. Crinnion, Sally M. Harrison, Carolina Lascelles, Agne Antanaviciute, Ian M. Carr, David T. Bonthron, Eamonn Sheridan

**Affiliations:** 1 Yorkshire Regional Genetics Service, St. James’s University Hospital, Leeds, LS9 7TF, United Kingdom; 2 School of Medicine, University of Leeds, St. James’s University Hospital, Leeds, LS9 7TF, United Kingdom; Virginia Tech, UNITED STATES

## Abstract

Next generation sequencing methodologies are facilitating the rapid characterisation of novel structural variants at nucleotide resolution. These approaches are particularly applicable to variants initially identified using alternative molecular methods. We report a child born with bilateral postaxial syndactyly of the feet and bilateral fifth finger clinodactyly. This was presumed to be an autosomal recessive syndrome, due to the family history of consanguinity. Karyotype analysis revealed a homozygous pericentric inversion of chromosome 7 (46,XX,inv(7)(p15q21)x2) which was confirmed to be heterozygous in both unaffected parents. Since the resolution of the karyotype was insufficient to identify any putatively causative gene, we undertook medium-coverage whole genome sequencing using paired-end reads, in order to elucidate the molecular breakpoints. In a two-step analysis, we first narrowed down the region by identifying discordant read-pairs, and then determined the precise molecular breakpoint by analysing the mapping locations of “soft-clipped” breakpoint-spanning reads. PCR and Sanger sequencing confirmed the identified breakpoints, both of which were located in intergenic regions. Significantly, the 7p15 breakpoint was located 523 kb upstream of *HOXA13*, the locus for hand-foot-genital syndrome. By inference from studies of HOXA locus control in the mouse, we suggest that the inversion has delocalised a *HOXA13* enhancer to produce the phenotype observed in our patient. This study demonstrates how modern genetic diagnostic approach can characterise structural variants at nucleotide resolution and provide potential insights into functional regulation.

## Introduction

Medical practitioners have, for many years, embraced new technologies in order to facilitate clinical diagnoses. Clinical genetics is an exemplar discipline in which technological advances have enabled continuously improving diagnostics, by providing finer-level information on the individual human genome. During the early 1960’s, the introduction of karyotyping enabled the detection of common aneuploidies and novel large structural abnormalities. The resolution of chromosome analysis was substantially increased following implementation of array-based technologies. However, the inability to detect balanced translocations and structural variations (such as inversions) was a potential weakness of this newer approach. In general, though, the increased diagnostic yield accruing from newer technologies more than compensates for individual examples of reduced sensitivity. Clinical genetics is currently on the fringe of a further transition resulting from the adoption of next-generation sequencing, with whole genome sequencing likely to become to the *de facto* frontline test in the not-too-distant future. Adoption of these methods is being influenced by the implementation of large-scale national initiatives in several countries.

In many diagnostic laboratories a step-wise approach towards whole genome sequencing has been adopted. This was initially by implementing analyses of small gene-panel tests [1], that have tended to be superseded by hybridisation capture enrichment of tens to thousands of genes [2]. In addition to single nucleotide and small insertion/deletion (indel) variant detection, focus has also been directed at detecting gross structural abnormalities using low coverage whole genome sequencing as an alternative to array comparative genome hybridiation (arrayCGH) [3]. Characterising structural variants at nucleotide resolution using medium coverage whole genome sequencing is a further step towards whole genome sequencing that has been successfully applied to diagnostic scenarios [4]. Here we show how a modification of our previously described workflow for medium coverage whole genome sequencing can allow nucleotide-level resolution of an unusual homozygous cytogenetic abnormality. In the process, we define a new mechanism through which a disorder usually displaying dominant inheritance can behave in a recessive fashion.

## Materials and Methods

Karyotyping was performed on the affected proband and her parents by the Yorkshire Regional Cytogenetics Laboratory. DNA was extracted from peripheral blood of the affected proband using a standard salting out method. Ethical approval was granted by the Leeds East Research Ethics Committee (07/H1306/113). The individual in this manuscript has given informed written consent (as outlined in the PLOS consent form) to publish these case details. Investigations were conducted according to the principles expressed in the Declaration of Helsinki.

### Breakpoint identification

An Illumina-compatible whole genome sequencing library was prepared using NEBNext^®^ Ultra^™^ reagents, following manufacturer’s protocols (New England Biolabs, Ipswich, MA, USA). Approximately 3 μg genomic DNA was sheared using a Covaris S2 (Covaris, Inc., Woburn, MA, USA) prior to undertaking sequential end-repair, dA addition and adaptor ligation reactions. The bead size selection ratio was for a 300-bp to 400-bp insert and a 6-cycle enrichment PCR was performed. The completed library was sequenced on a single lane of a paired-end HiSeq2500 rapid flow cell setup to generate asymmetric read pairs of lengths 175 bp and 50 bp. The raw data were demultiplexed using CASAVA v.1.8.2 and the resulting FASTQ.gz files were run through an in-house data processing pipeline (these data are available from the European Nucleotide Archive using study accession number PRJEB13759). Adaptor sequences were trimmed from the ends of the sequence reads using Cutadapt v.1.7.1 (https://code.google.com/p/cutadapt/) [5] before read alignment to the human genome (hg19) using bwa v.0.7.12 (http://bio-bwa.sourceforge.net) [6]. Picard v.1.129 (http://broadinstitute.github.io/picard/) was used to perform standard sam file manipulation tasks.

Sequence reads mapping to 7p15 (chr7:20900001–28800000) were extracted from the coordinate-sorted duplicate-marked bam file using samtools v.0.1.18 (http://samtools.sourceforge.net) [7], specifying that the mapping quality score was greater than 0, neither read in the pair was unmapped and the pair was not considered to be a “proper pair”. These reads were selected using samtools options -q 1 and -F 14. The resulting read pairs were filtered to retain only those whose mate read mapped to 7q21 (chr7:775000001–98000000). The coordinate-sorted order of read pairs was maintained and those read pairs whose mate mapped within 1 kb of the preceding read were retained and further examined using the Integrative Genomics Viewer (IGV) (https://www.broadinstitute.org/igv/) [8]. Soft-clipped putatively breakpoint-spanning reads were interrogated using BLAT (http://genome.ucsc.edu/cgi-bin/hgBlat) [9].

### Molecular assay enabling breakpoint confirmation

PCR amplicons were designed to amplify across the identified breakpoints. The primers used to amplify the 7p15 breakpoint were dTGTAAAACGACGGCCAGTGCCCGGCTAATTCACACAAT (common 7p15 forward) and dCAGGAAACAGCTATGACCTGGTCTTCACAGAGAGAGTATCA (reverse 7q21 inversion), which generated a 395-bp PCR product. A second reverse primer (dCAGGAAACAGCTATGACCGCCATTAACACACCACCCAA) was designed to work in combination with the common forward primer to generate a 438-bp PCR product specific for the normal 7p15 allele. The primers used to amplify the 7q21 breakpoint were dTGTAAAACGACGGCCAGTGCCATTAACACACCACCCAA (forward 7p15 inversion) and dCAGGAAACAGCTATGACCCCCAATGTTCCAGGGGCTA (common 7q21 reverse), which generated a 359-bp product. A second forward primer (dTGTAAAACGACGGCCAGTTGCCCTACTTGGTCTCATTGA) was designed to work in combination with the common reverse primer to generate a 492-bp PCR product specific for the normal 7q21 allele. Each PCR consisted of 0.5 μl of genomic DNA (250 ng/μl), 11 μl of MegaMix (Microzone Ltd., Haywards Heath, UK), 1 μl of 10 pmol/μl (7p15 amplicons) or 20 pmol/μl (7q21 amplicons) forward primer and 1 μl of 10 pmol/μl (7p15 amplicons) or 20 pmol/μl (7q21 amplicons) reverse primer. Each primer contained a universal tag (underlined) allowing Sanger sequencing according to our standard laboratory workflow. Thermocycling conditions consisted of 94°C for 5 minutes, 30 cycles of 94°C for 30 seconds, either 55°C (7p15 amplicons) or 53°C (7q21 amplicons) for 1 minute and 72°C for 45 seconds before a final extension step at 72°C for 5 minutes. Sanger sequencing on an ABI3730 confirmed the identity of all PCR fragments, manufacturer’s protocols were followed throughout (Life Technologies Ltd., Paisley, UK). Sequence chromatograms were analysed using Chromas Lite v.2.1.1 (http://www.technelysium.com.au) and are provided as supplementary material.

## Results

### Clinical description

**Fig 1:**
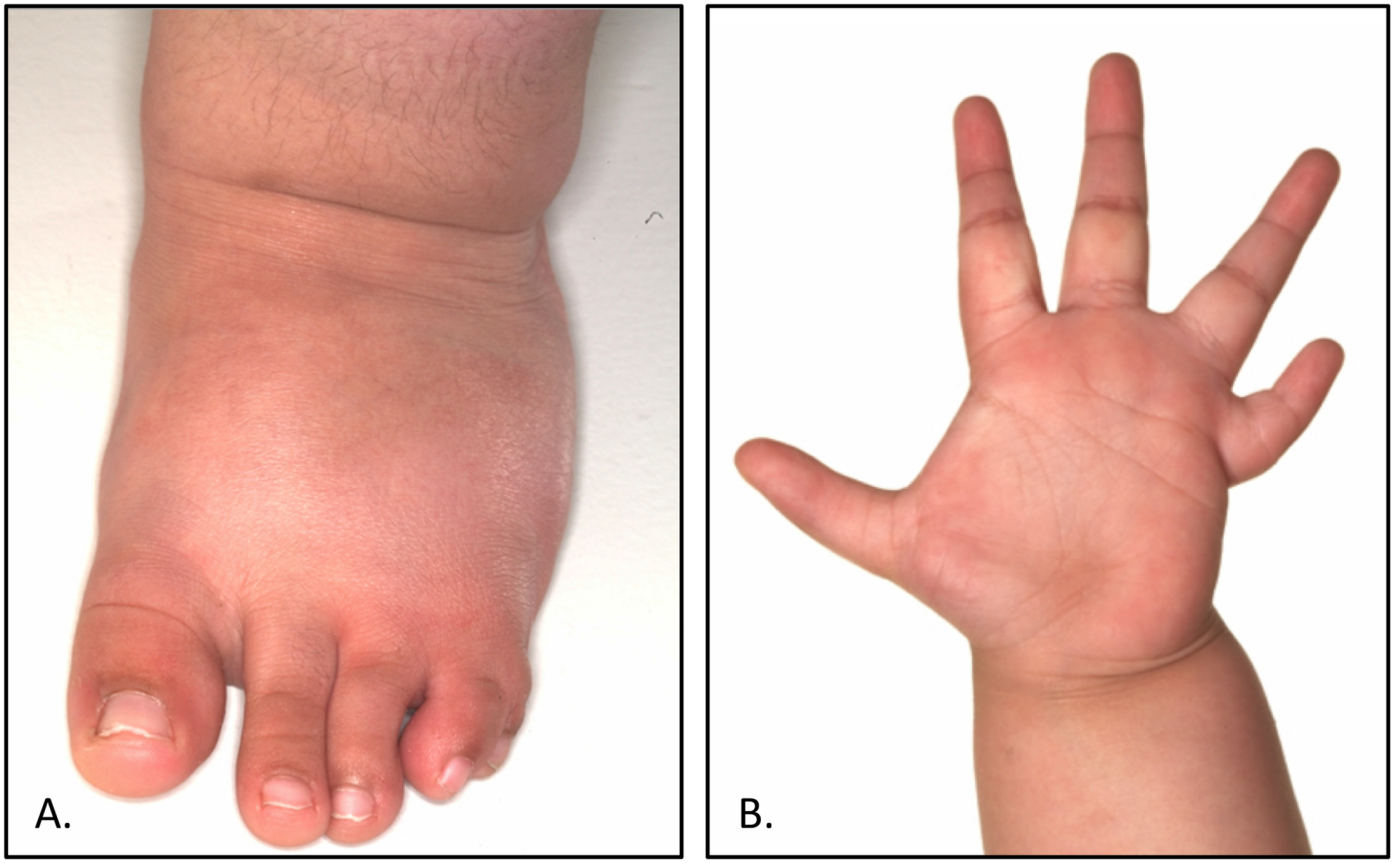
Clinical photos. **(A)** Left foot showing syndactyly of fourth and fifth toes. **(B)** Left hand showing proximally placed thumb and marked clinodactyly of fifth finger.

The patient was the first child of consanguineous parents. She was born following a normal pregnancy at term with a birth weight of 2.8 kg. In the newborn period she was noted to have short stubby feet with bilateral postaxial syndactyly, marked clinodactyly of the fifth fingers bilaterally and proximally placed thumbs, with hypoplasia of the thenar eminence (Fig 1). There has been no history of urinary tract problems and contrast-enhanced imaging of the urinary tract was normal. The parents did not consent to gynaecological examination of their daughter; external examination of the genitalia was normal. Abdominal ultrasound confirmed the presence of a normally sited uterus.

### Cytogenetic analysis

Karyotyping revealed a homozygous pericentric inversion in the affected child with breakpoints in cytogenetic bands 7p15 (chr7:20,900,001–28,800,000) and 7q21 (chr7:77,500,001–98,000,000), which span 7.9 Mb and 20.5 Mb respectively (S1 Fig). Parental karyotyping revealed that both parents were heterozygous carriers of the balanced chromosome 7 pericentric inversion, indicating that this variant had not arisen *de novo*.

### Whole genome sequencing

To determine the molecular breakpoints of the inversion, medium coverage (~9×) whole genome sequencing was performed using asymmetric (175-bp and 50-bp) paired-end sequencing reads. Approximately 225 million reads were aligned to the human reference genome (S1 Table) and the aligned bam file was interrogated to identify discordant read pairs (those with a larger than expected insert size) mapping to both 7p15 and 7q21, yielding 609 read pairs. These read pairs were further filtered to identify mates which were mapped less than 1 kb from the previous read pair and had a mapping quality score >0 (thus excluding reads that mapped to multiple locations). One cluster comprising four such read pairs was identified (Table 1). The mate-pair sequences mapped within 100 bp of each other and the apparent insert size defined by these read pairs was ~66 Mb (consistent with the size of the inverted chromosome 7 segment). Both reads in the pair mapped to the same strand, as expected for a true structural variant. Furthermore, the 175-bp read of each pair had been “soft-clipped”, due to a mismatch between the read and the reference sequence. Visualisation of the aligned bam file in the IGV browser revealed a further two soft-clipped reads located at the putative 7q21 breakpoint. Following BLAT mapping of all six identified 175-bp reads, it was apparent that the soft-clipped sequence spanned the chromosome 7 inversion breakpoint (Table 2).

**Table 1:** Reads pairs with discordant reads mapping to 7p15 and 7q21 within 200 bp of each other.

		Read 1				Read 2				
Read pair index	Read pair ID	Position	Strand	MAQ	CIGAR	Position	Strand	MAQ	CIGAR	Apparent insert size (bp)
1	HWI-ST1297:143:H8A5HADXX:2:1211:13013:74367	27,762,167	+	6	50M	93,599,431	+	60	102M73S	65,837,265
2	HWI-ST1297:143:H8A5HADXX:2:2216:6929:62067	27,762,258	+	53	50M	93,599,409	+	60	124M51S	65,837,152
3	HWI-ST1297:143:H8A5HADXX:2:2106:11510:54958	27,762,316	+	60	110M65S	93,599,416	+	60	50M	65,837,101
4	HWI-ST1297:143:H8A5HADXX:2:1205:16877:58763	27,762,326	+	60	100M75S	93,599,338	+	60	50M	65,837,013

MAQ: Mapping quality score; M: Matched nucleotides; S: soft-clipped nucleotides

**Table 2:** Characteristics of soft-clipped breakpoint-containing reads.

Read pair index	Read pair ID	Sequence	Aln (nts)	Sc (nts)	Aln start	Aln end	Aln str	Sc start	Sc end	Sc str
1	1211:13013:74367	AAGATGTTTTACTCTGCAATAAAATTTTGTACAATCTAACATTCATGAAAGAGGGAAATAAGTGGATTTTGCAAAGAATATTCACCAAGACCTTTGTTTAATTTAAAATAGTCTTCTGCAACTTGATTTTGTTCACCCAAGGGTATGTTTTCAAGATTCATCTATATTGTTGCTT	102	73	93,599,431	93,599,532	+	27,762,423	27,762,351	-
2	2216:6929:62067	TTACATTAGAGTATTTGCATTAAAGATGTTTTACTCTGCAATAAAATTTTGTACAATCTAACATTCATGAAAGAGGGAAATAAGTGGATTTTGCAAAGAATATTCACCAAGACCTTTGTTTAATTTAAAATAGTCTTCTGCAACTTGATTTTGTTCACCCAAGGGTATGTTTTCA	124	51	93,599,409	93,599,532	+	27,762,423	27,762,373	-
3	2106:11510:54958	ACAGCAGTGAAAATGAATGTGTGAGCTACAGCTACAAGCAACAATATAGATGAATCTTGAAAACATACCCTTGGGTGAACAAAATCAAGTTGCAGAAGACTATTTTAAATTAAACAAAGGTCTTGGTGAATATTCTTTGCAAAATCCACTTATTTCCCTCTTTCATGAATGTTAG	110	65	27,762,316	27,762,425	+	93,599,530	93,599,466	-
4	1205:16877:58763	AAATGAATGTGTGAGCTACAGCTACAAGCAACAATATAGATGAATCTTGAAAACATACCCTTGGGTGAACAAAATCAAGTTGCAGAAGACTATTTTAAATTAAACAAAGGTCTTGGTGAATATTCTTTGCAAAATCCACTTATTTCCCTCTTTCATGAATGTTAGATTGTACAAA	100	75	27,762,326	27,762,425	+	93,599,530	93,599,456	-
5	1102:3940:94914	GCATTAAAGATGTTTTACTCTGCAATAAAATTTTGTACAATCTAACATTCATGAAAGAGGGAAATAAGTGGATTTTGCAAAGAATATTCACCAAGACCTTTGTTTAATTTAAAATAGTCTTCTGCAACTTGATTTTGTTCACCCAAGGGTATGTTTTCAAGATTCATCTATATTG	108	67	93,599,425	93,599,532	+	27,762,423	27762357	-
6	1215:19689:22918	GGGAAATAAGTGGATTTTGCAAAGAATATTCACCAAGACCTTTGTTTAATTTAAAATAGTCTTCTGCAACTTGATTTTGTTCACCCAAGGGTATGTTTTCAAGATTCATCTATATTGTTGCTTGTAGCTGTAGCTCACACATTCATTTTCACTGCTGTATAAAATTCTATTGTGT	50	125	93,599,483	93,599,532	+	27,762,423	27,762,299	-

Matching nucleotides are underlined; Aln: Aligned; Sc: Soft-clipped; str: strand

We have previously described a diagnostic split read mapping workflow to identify breakpoint-spanning reads among those reads that fail to align to the reference genome [4]. In the present case, the split-read mapping workflow did not yield any additional breakpoint-spanning reads.

### Breakpoint confirmation

**Fig 2:**
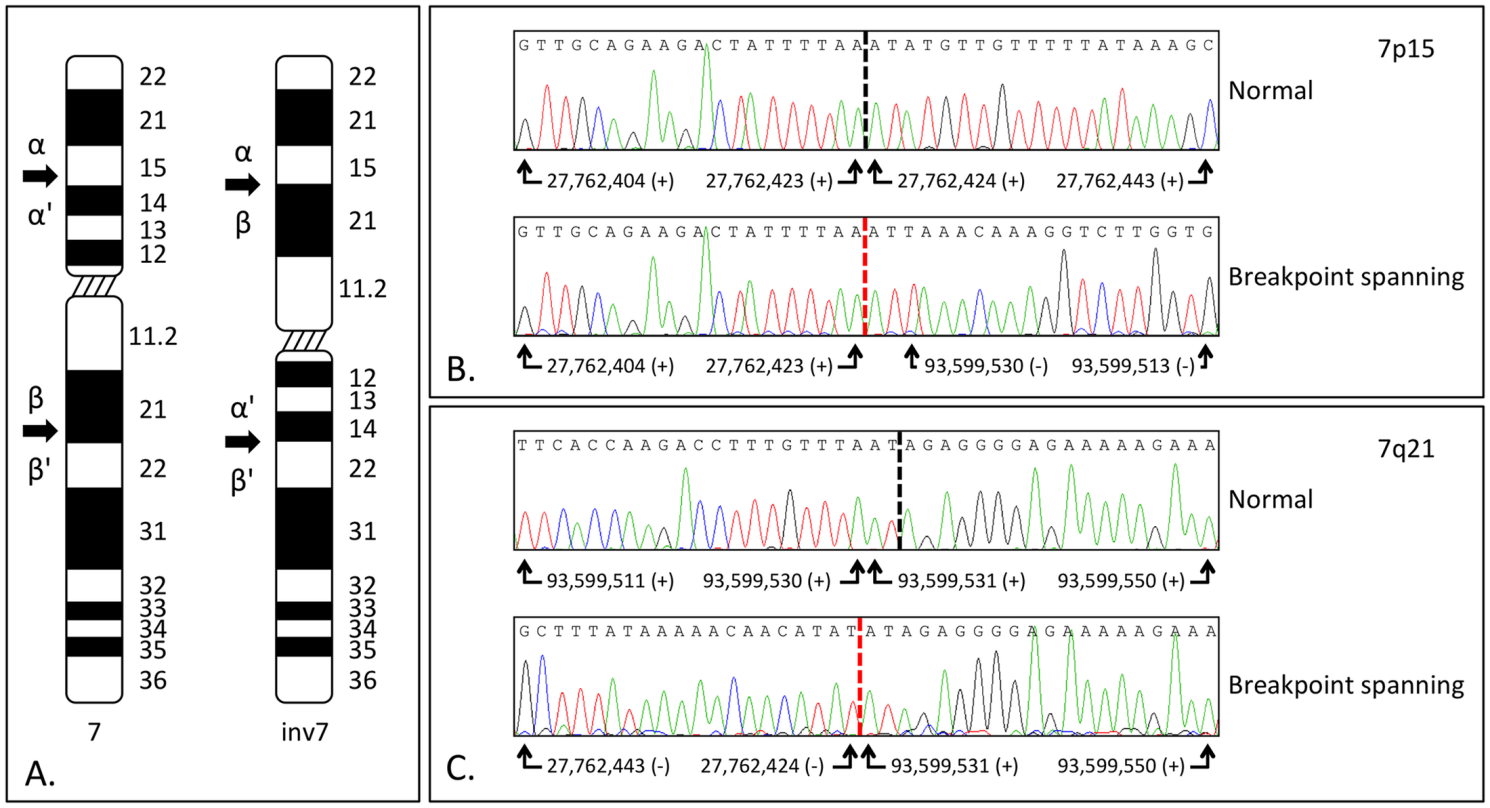
Chromosome 7 ideogram and breakpoint confirmation. **(A)** Arrows showing the breakpoint locations. Greek letters facilitate interpretation of the resulting pericentric inversion. Sanger sequencing results for the normal and breakpoint spanning amplicons for **(B)** the 7p15 and **(C)** the 7q21 inversion boundaries. The vertical dashed read line highlights the breakpoint. For ease of comparison a dashed black line has been drawn onto the normal sequence. (+): sense strand sequence; (-): antisense strand sequence. The inversion has resulted in an AT dinucleotide duplication which is shown arbitrarily assigned to the 7p15 breakpoint.

To validate the inversion coordinates, breakpoint-spanning PCRs were designed. Sanger sequencing of these amplicons confirmed the inversion, which included an AT duplication which can be arbitrarily assigned to be at either the 7p15 or 7q21 breakpoint, due to local microduplication in the normal 7p sequence (Fig 2). These breakpoints were positioned at hg19 coordinates 27,762,423–4 and 93,599,530–1, respectively. Both of these positions are intergenic, so that it does not appear that physical transection of a coding region accounts for the phenotype observed in this family. However, we noted that the chromosome 7p15 breakpoint lies approximately 523 kb upstream of *HOXA13*, the gene that is mutated in autosomal dominant hand-foot-genital syndrome (OMIM: 140000). This breakpoint lies in the intergenic region between *HIBADH* and *TAX1BP1* (neither of which is an OMIM morbid gene), in a region known to be important for long-range control of the *Hoxa* gene cluster in the developing mouse limb [10].

## Discussion

The complexity of genetic diagnostics is reduced in individuals with a family history of consanguinity by focussing analyses on regions of the genome that are identical by descent. In this regard autozygosity mapping and candidate gene screening to identify single nucleotide and small indel variants has led to the identification of many new disease genes. Structural variants identified by karyotyping or more recently arrayCGH are also amenable to this approach [4].

Whether homozygous or heterozygous, an apparently balanced chromosomal rearrangement may result in a phenotype that usually displays a Mendelian inheritance pattern, because the rearrangement transects a causative gene. However, rearrangements may also be associated with cryptic aneuploidy, so molecular analysis is needed in order to confirm or refute the involvement of a single gene.

In the present case, we initially hypothesized that the homozygous rearrangement (secondary to parental consanguinity) had unmasked a recessive disease-causing allele. Molecular characterisation using whole genome sequencing determined that both chromosome 7 breakpoints were located in intergenic regions. However, a review of the literature concerning characterised disorders known to map near these breakpoints highlighted hand-foot-genital syndrome (HFGS; OMIM: 140000). HFGS is characterised by fully penetrant limb abnormalities and incompletely penetrant urogenital defects and is caused by heterozygous mutations in *HOXA13*. The original basis for investigating *HOXA13* as a candidate gene for HFGS was the identification of a spontaneous frameshifting deletion in *Hoxa13* exon 1 of the *Hypodactyly* mouse [11]. The heterozygous mutant mice have limb abnormalities including short first digits with small distal phalanges, hypoplastic second and fifth middle phalanges and delayed bone ossification [12]. Perhaps significantly (given the variable expressivity of urogenital features in humans) the mice lacked urogenital tract abnormalities. The first variant reported to cause human HFGS was the c.1107G>A (p.Trp369X) nonsense mutation [13]. At least 17 different mutations have since been reported, with coding sequence variants accounting for ~35% of cases. Most frequently, the causative pathogenic variant is an expansion of one of three polyalanine tracts located in the first exon of *HOXA13* (~50–60% of cases) [14]. The chromosome 7p15 breakpoint reported here lies 523 kb upstream of *HOXA13*.

*HOXA13* is a member of the *HOXA* gene cluster, one of four HOX gene clusters that together comprise 39 mammalian genes and are of central importance to body patterning during embryonic development. Previous studies of the *HoxD* cluster have shown regulation to be controlled by long-distance enhancers [15]. Initial data suggesting that the *HoxA* cluster could be regulated by long-range enhancers was provided by BAC transgenesis experiments, which demonstrated that two regions in the vicinity of *Hibadh* and remote from the HoxA cluster were required to drive Hoxa13-like expression [16]. Subsequent results obtained from mouse distal limb cells determined that expression of *HoxA* genes is controlled by multiple enhancers located 5′ of the cluster; a number of the *HoxA* identified enhancers are located beyond the syntenic human 7p15 inversion breakpoint we describe [10] (S2 Fig). The previously described enhancers were proposed to form sub-megabase topological domains that contact both themselves and target gene-containing domains (as opposed to interacting as discrete loops between themselves and their target gene). Although these experiments were reported in mice, an analysis of the human genome noted that *Hox* gene clusters contain the lowest density of interspersed repeats, an observation attributed to the essential requirement to conserve the arrangement of genes and *cis*-acting regulatory elements [12]. Our observation raises the possibility that a defect in the spatial or temporal expression of *HOXA13* may be giving rise to the phenotype observed in the reported case, since the inversion would act to delocalise the reported enhancer sequences from the HoxA cluster.

HFGS has been previously described as an autosomal dominant entity with 100% penetrance [14]. Previous studies reporting small interstitial deletions have demonstrated that loss of a single copy of *HOXA13* is sufficient to cause HFGS and have led to the proposal that haploinsufficiency is the primary mechanism of *HOXA13* pathogenesis [17–18], data that was supported by transgenic mouse models [19]. Our finding that heterozygous carriers of the 7p15q21 inversion have no phenotype suggests that any long-range-position effect on HOXA13 is markedly weaker than the *HOXA13* haploinsufficiency that has been previously described.

The role of HOXA13 in the developing limb has been largely elucidated through analyses of mutant mice. These studies have revealed how the HOXA13 transcription factor, which binds AT-rich DNA sequences, regulates downstream targets *Bmp2*, *Bmp7*, *EphA7* and *Sostdc1*, which are necessary for apoptosis and cell sorting in the developing autopod [20]. While nonsense mutations and polyalanine tract expansions are thought to result in loss of function alleles, pathogenic missense mutations in the homeodomain appear to confer a gain of function, which is associated with a more severe limb phenotype [21].

Here we report the use of an asymmetric paired-end sequencing run configuration to resolve a pericentric inversion at nucleotide resolution. Our approach allowed us to first identify discordant read pairs to focus our analysis on a reduced genomic location, before we identified “soft-clipped” reads that contained breakpoint-spanning sequence. When applying short-read next generation sequencing technology to the identification of structural variants, success depends on the underlying genomic configuration. Highly repetitive sequences, which are prone to mutation due to non-allelic homologous recombination, are often difficult to sequence. Increasing read lengths will likely go some way to overcoming this limitation.

We have previously described a workflow to identify breakpoint spanning reads by performing split-read alignments, using the pool of reads that fail to map to the reference genome [4]. As next-generation sequencing alignment algorithms become progressively better at mapping reads on the basis of partial matches, the proportion of unmapped reads will diminish. A more appropriate strategy to resolve structural variants may therefore be to use asymmetric read-pair sequencing to identify discordant read-pairs with soft-clipped reads.

Novel genetic insights are typically confirmed following identification of cases with similar phenotypes in which variants occur in the same gene or overlapping genomic regions. For structural variants including deletions and duplications the coordinates of variant breakpoints are frequently unique. To overcome the challenge of querying these variants, tools such as DECIPHER provide an-easy-to-use graphical viewer [22]. In addition these tools represent a community resource cataloguing genomic variants that can be accessed by the medical professionals. With the establishment of many national and international large-scale medical sequencing projects, these tools will become increasingly important. Despite their utility, the ability to record and visualise less common classes of variant (such as inversions) is frequently restricted. It may therefore be that despite ongoing sequencing efforts the ability to identify similar families with rare classes of structural variation will lag behind that of SNV and small indel detection for some time to come. Thus, to-date, the inversion we describe remains unique and we have been unable to locate, in public databases, any other example of an HFGS phenotype resulting from *HOXA13* delocalisation from the enhancer elements. Should our observation be confirmed in additional cases it will be the first association of a chromosome 7 inversion with HFGS.

Our study is also limited by the structure of the investigated pedigree (with only a single affected family member). Furthermore, a lack of patient material and the unknown temporal expression profile of HOXA13 in humans precluded any analysis of the effect on *HOXA13* transcription *in vitro*. Emerging technologies such as the CRISPR/Cas9 targeted genome editing tools may, in future years, make cellular modelling of presumptive regulatory variants a realistic endeavour. Beyond the technical challenges posed by these experiments, biological insight will be required to ensure appropriate cell types development time points are selected. At present, the pathogenicity of the described variant is supported by inference from studies of the murine Hoxa cluster which displays conserved synteny with the human HoxA locus. It remains possible that the Chr.7 inversion may be affecting the regulation of a gene not previously associated with HFGS. For example, in addition to *Hoxa13*, *Evx1*, *Hibadh*, *Tax1bp1* and *Jazf1*, have all been demonstrated to have distal autopod and genital bud expression from E10.5 through to E13.5 [23]. Although our experiments were accomplished in a clinical diagnostic laboratory, the cost and equipment requirements of whole genome sequencing may hinder the wider implementation of our method. Nevertheless, as the cost of sequencing continues to fall, and large-scale, national whole genome sequencing projects continue to be announced, appropriate datasets to apply our reported methodology will become increasingly prevalent.

Our study highlights how variants identified using traditional cytogenetic methods can be characterised at the molecular level using next-generation sequencing in combination with bespoke analyses. Indeed, had the standard frontline test of arrayCGH been undertaken in absence of karyotyping, this variant would not have been detected. The precise molecular characterisation of variants such as the pericentric inversion described will likely yield further novel insights into human biology.

## Supporting Information

S1 Fig Patient karyogram. Arrows depict chromosome 7 inversion breakpoints.
(TIF)

S2 Fig Region of conserved synteny between human and mouse loci at the proximal end of the HoxA cluster. The RefSeq Gene track is displayed for **(A)** human reference genome build hg19 and **(B)** mouse reference genome build mm10.
(TIF)

S1 File 7p15 amplicon sequenced from a normal unaffected control. (AB1)

S2 File 7p15 breakpoint spanning amplicon. (AB1)

S3 File 7q21 amplicon sequenced from a normal unaffected control. (AB1)

S4 File 7q21 breakpoint spanning amplicon. (AB1)

S1 Table Sequencing and alignment metrics. (DOCX)
