# Supplementary figures and images for: A Chromosome 7 Pericentric Inversion Defined at Single-Nucleotide Resolution Using Diagnostic Whole Genome Sequencing in a Patient with Hand-Foot-Genital Syndrome
Source: PLoS One. 2016 Jun 7;11(6):e0157075. doi: 10.1371/journal.pone.0157075 (PMC4896502; doi:10.1371/journal.pone.0157075)

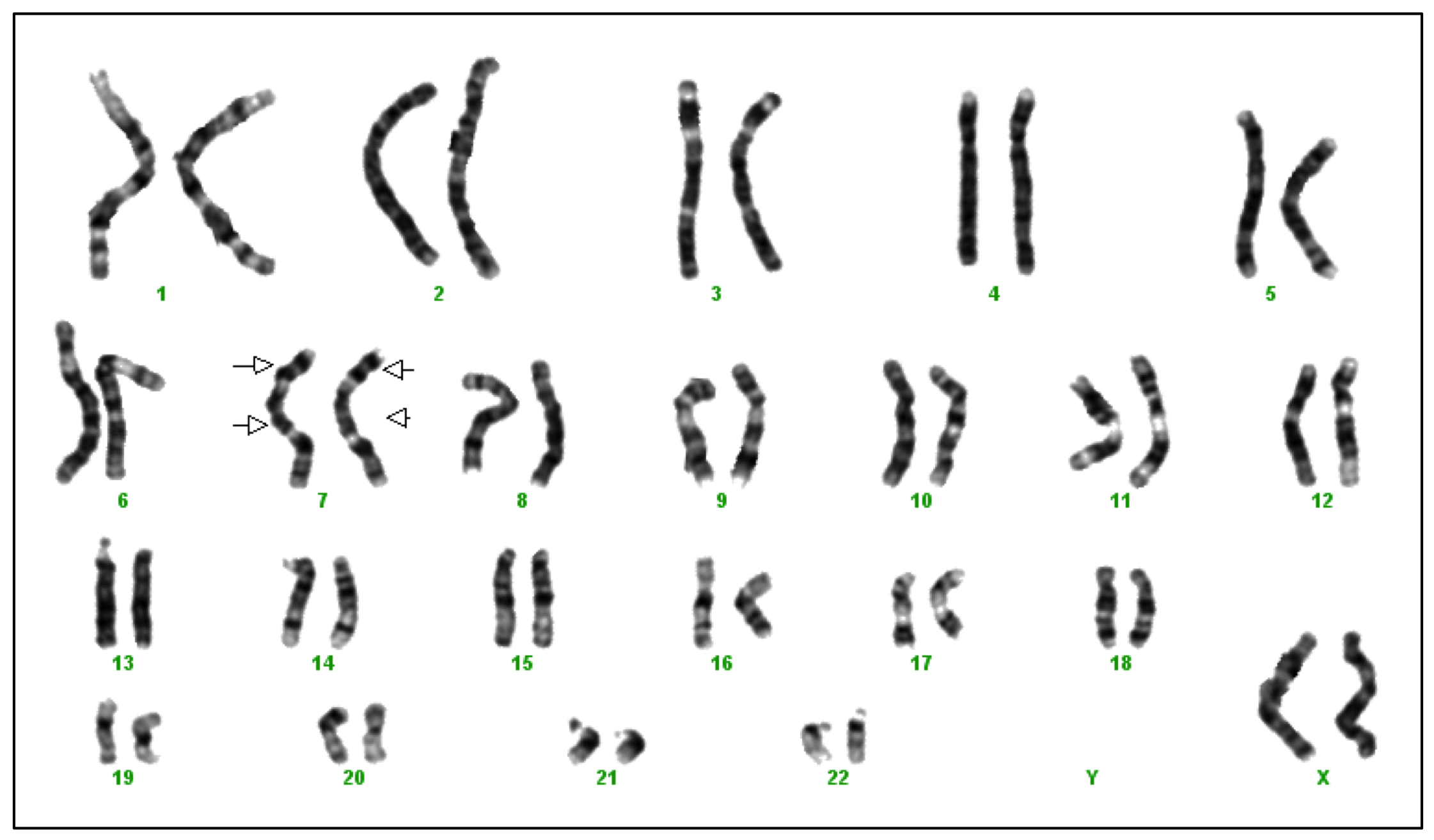

Supplement: S1 Fig — Arrows depict chromosome 7 inversion breakpoints. (TIF) [file pone.0157075.s001.tif]

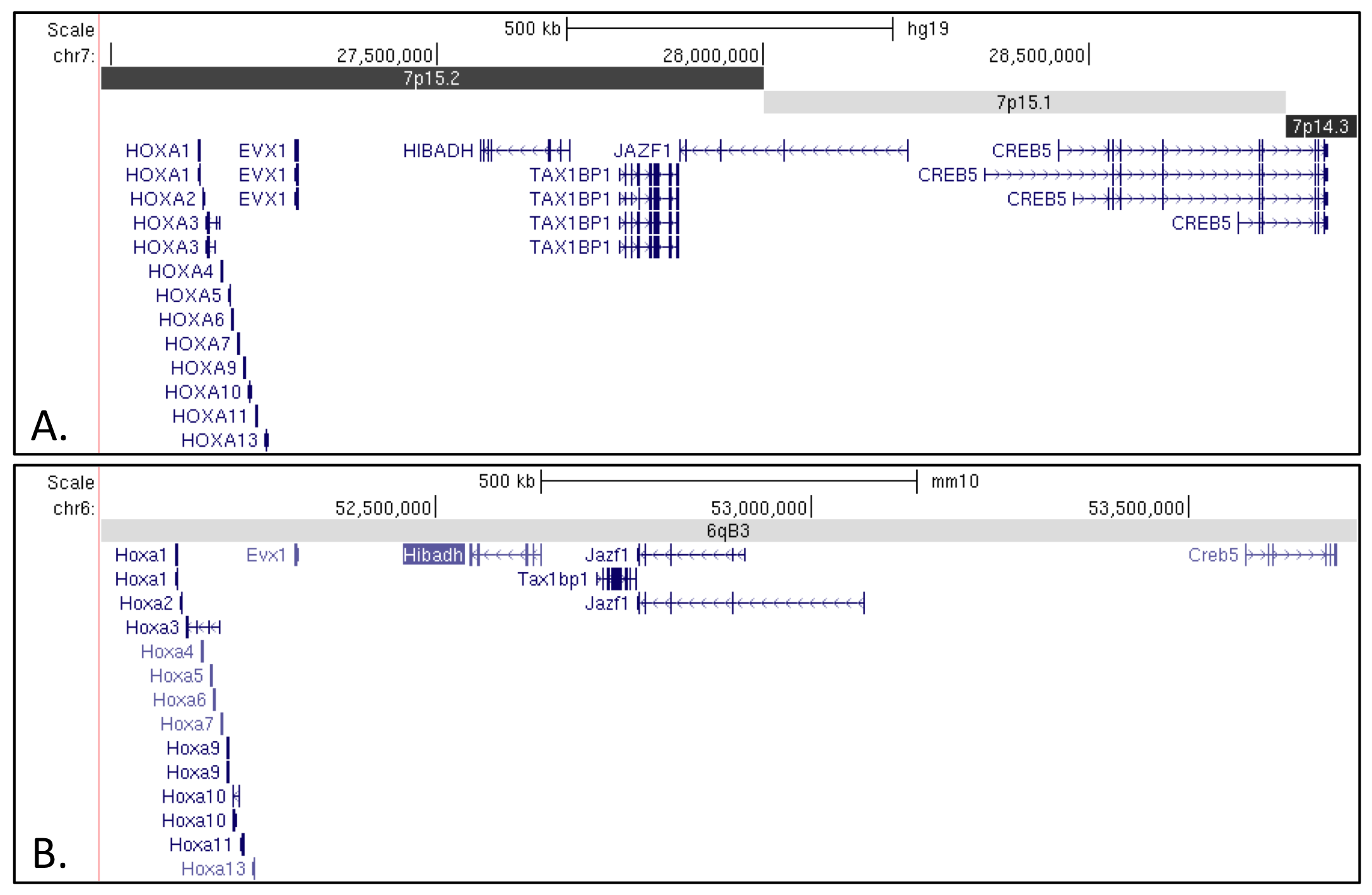

Supplement: S2 Fig — The RefSeq Gene track is displayed for (A) human reference genome build hg19 and (B) mouse reference genome build mm10. (TIF) [file pone.0157075.s002.tif]
