# Supplementary material for: A Chromosome 7 Pericentric Inversion Defined at Single-Nucleotide Resolution Using Diagnostic Whole Genome Sequencing in a Patient with Hand-Foot-Genital Syndrome
Source: PLoS One. 2016 Jun 7;11(6):e0157075. doi: 10.1371/journal.pone.0157075 (PMC4896502; doi:10.1371/journal.pone.0157075)
Supplement: S1 Table — (DOCX) [file pone.0157075.s007.docx]

**S1 Table. Sequencing and alignment metrics.**

| **Read length configuration** | **Total raw reads** | **Adaptor trimmed reads** | | **Duplicate rate (%)** | **Mean insert size (bp)** | **Total mapped reads** |
| --- | --- | --- | --- | --- | --- | --- |
|  |  | **Read 1 (%)** | **Read 2 (%)** |  |  |  |
| 175 bp and 50 bp read lengths | 236,713,972 | 11.4 | 2.7 | 1.68 | 264 | 226,877,151 |
